# Renal Cell Carcinoma Metastasizing to the Myocardium: A Comprehensive Literature Review of Diagnostic and Therapeutic Challenges

**DOI:** 10.7759/cureus.99988

**Published:** 2025-12-24

**Authors:** Ayham Khan Ansari, Ibrahim Ibrar, Ans A Mahmood, Sarah I Zahid, Muhammad Kamil Shahbaz, Jayakumary Muttappallymyalil

**Affiliations:** 1 Internal Medicine, College of Medicine, Gulf Medical University, Ajman, ARE; 2 Public Health, Gulf Medical University, Ajman, ARE

**Keywords:** cardiac metastasis, cardiac mri, immunotherapy, inferior vena cava, myocardium, renal cell carcinoma, tyrosine kinase inhibitors

## Abstract

Renal cell carcinoma is a type of cancer that occurs in adults and typically metastasizes to the lungs, liver, bone, and brain. Cardiac involvement is far less common and often goes unrecognized, as its clinical manifestations can mimic those of more prevalent cardiopulmonary conditions, leading to substantial morbidity. This review examines the epidemiology, mechanisms of spread, patterns of presentation, diagnostic approaches, and treatment strategies for renal cell carcinoma involving the myocardium. Mechanisms include direct extension through the inferior vena cava to the right atrium, hematogenous dissemination, and, less frequently, lymphatic spread. Clinical findings range from incidental imaging abnormalities to presentations involving heart failure, arrhythmias, or cardiac tamponade. Diagnosis relies on multimodal imaging, with echocardiography and cardiac MRI providing complementary information. Management options include surgery in selected cases and systemic therapies, although available evidence is mainly derived from case reports and small series. Overall outcomes remain poor. Early recognition, coordinated multidisciplinary care, and further research are needed to guide management in this rare clinical setting.

## Introduction and background

Renal cell carcinoma represents a modest proportion of adult malignancies, yet a substantial number of patients present with metastatic disease at diagnosis [1]. Although the lungs, bone, liver, and brain comprise the most frequent sites of distant metastasis, cardiac involvement is distinctly uncommon and usually accompanies widespread malignancy [1,2]. Autopsy studies have consistently demonstrated higher rates of cardiac metastases than those identified clinically, suggesting that many lesions remain asymptomatic and undetected during life [3].

Among the published cases, the pericardium is the most frequently affected cardiac structure, followed by the myocardium and endocardium [3,4]. Clear cell carcinoma accounts for most reported cases and is known for its aggressive vascular behavior and ability to invade venous structures [4,5]. The anatomical relation between the renal vein, inferior vena cava, and right atrium explains why right-sided involvement is common [3,6].

Clinical features often mimic common cardiac or pulmonary conditions, contributing to delayed diagnosis. Imaging plays a central role, with transthoracic echocardiography and cardiac MRI serving as the primary modalities for evaluating suspected cardiac involvement [3,6,7]. Because standardized management guidelines are lacking, treatment is individualized. This review synthesizes current evidence regarding the mechanisms, clinical implications, and management of myocardial metastases from renal cell carcinoma.

## Review

Epidemiology

Cardiac metastases arising from renal cell carcinoma are rare, and determining true incidence is challenging due to reliance on isolated case reports and small retrospective series [1-3]. Autopsy findings indicate a somewhat higher prevalence, suggesting that many cases remain clinically silent [3]. Cardiac involvement is typically seen in patients with advanced or recurrent disease. Clear cell carcinoma predominates in the literature, aligning with its highly angiogenic biology and propensity for venous invasion [3-6].

Although right-sided cardiac structures are most commonly affected, reflecting expected venous pathways, metastases involving left-sided chambers or multiple cardiac regions have also been described [1,3,6,7]. The limited number of systematically collected cases makes it difficult to accurately define risk factors or estimate true prevalence.

Mechanisms of spread

**Figure 1:**
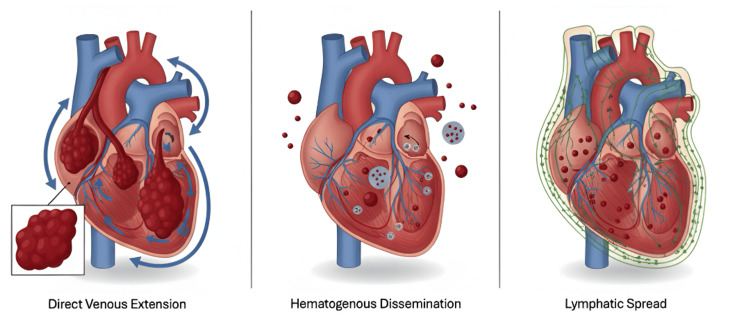
Mechanisms of metastatic spread to the heart The illustration demonstrates three major metastatic pathways: direct venous extension through the renal vein and inferior vena cava to the right atrium, hematogenous dissemination resulting in intramyocardial deposits, and lymphatic spread associated with pericardial involvement. This schematic figure was created by the authors using Google Gemini (image generation; Google, Mountain View, CA, USA) and Microsoft PowerPoint (annotation and layout; Microsoft Corporation, Redmond, WA, USA).

Cardiac metastases from renal cell carcinoma occur through three main pathways: direct venous extension, hematogenous dissemination, and lymphatic spread. These mechanisms differ in their anatomical involvement and clinical implications (Figure 1).

Direct Venous Extension

Direct tumor extension from the renal vein into the inferior vena cava and right atrium remains the most extensively documented mechanism. Imaging and operative findings have repeatedly confirmed this pathway, particularly in patients with bulky or obstructive intracaval thrombus [1,3,7]. Smaller or asymptomatic intravascular extensions are likely underrecognized.

Hematogenous Dissemination

Some patients develop isolated intramyocardial deposits without inferior vena cava involvement, suggesting hematogenous spread. This appears especially relevant in clear cell renal carcinoma, where von Hippel-Lindau-associated angiogenesis may promote dissemination [3-6,8]. Reports of delayed cardiac recurrence years after nephrectomy support the concept of dormant micrometastatic disease [8,9].

Lymphatic Spread and Delayed Metastasis

Lymphatic dissemination has been proposed in cases involving the pericardium without venous extension. Although plausible, this pathway is less clearly demonstrated because dedicated lymphatic imaging and autopsy confirmation are limited [2,10].

Clinical presentation

Clinical presentations vary widely. Some patients are asymptomatic, with lesions detected incidentally on surveillance imaging, while others exhibit exertional dyspnea, chest discomfort, palpitations, fatigue, or syncope. When right-sided chambers are affected, systemic venous congestion may manifest with peripheral edema or hepatic enlargement [1,6,8-10].

Myocardial infiltration may disrupt conduction pathways, giving rise to atrial or ventricular arrhythmias. Pericardial metastases can produce effusion or tamponade, which may present abruptly and require urgent intervention [10,11]. Because findings overlap with common cardiopulmonary disorders, diagnosis often depends on a high index of suspicion in patients with known malignancy.

Diagnostic evaluation

Diagnosis relies on multimodal imaging. Transthoracic echocardiography is often the initial modality because it is readily available and capable of demonstrating intracardiac masses, venous obstruction, and pericardial effusion [1-3,6]. However, its sensitivity is limited for small or intramyocardial lesions.

Cardiac MRI provides superior tissue characterization and enables differentiation between tumor and thrombus while defining the extent of myocardial involvement [3-6,8]. Computed tomography is valuable for assessing inferior vena cava involvement and systemic disease burden, whereas positron emission tomography can identify metabolically active lesions, though uptake in renal cell carcinoma is variable [4-6,8]. Despite consistent use of these imaging tools, no standardized diagnostic pathways exist.

Management strategies

Because formal guidelines are lacking, treatment is individualized. Surgical resection may be appropriate for selected patients with isolated or obstructive intracardiac lesions and adequate functional reserve. In such cases, surgery may relieve symptoms or improve hemodynamics [1,3,7,12]. However, operative risks are significant, and reported cases typically involve carefully chosen candidates.

Systemic therapy is central to managing metastatic renal cell carcinoma. Immune checkpoint inhibitors and tyrosine kinase inhibitors have been used for patients with cardiac involvement, with some reports describing radiographic stabilization. Nonetheless, these agents carry potential cardiotoxicity, including myocarditis, arrhythmias, hypertension, and heart failure, which complicates decision-making in affected patients [4-6,8-11]. For individuals with extensive metastatic disease or limited physiological reserve, care is largely supportive and focuses on symptom management, heart failure optimization, and control of arrhythmias or pericardial effusions.

Themes, trends, and gaps in the literature

Across published reports, several consistent themes emerge. Cardiac metastases occur across a broad range of clinical scenarios and may appear many years after nephrectomy, demonstrating the unpredictable nature of metastatic renal cell carcinoma. Imaging plays a central role, but approaches vary widely, reflecting the absence of standardized diagnostic algorithms.

Significant gaps persist. Natural history, prognostic indicators, and the influence of tumor biology on cardiac tropism remain poorly understood. Criteria for selecting patients for surgery are unclear, and the safety of systemic therapies in those with cardiac involvement requires further study. Larger multicenter registries and prospective data collection are needed to better characterize disease patterns and guide management.

## Conclusions

Cardiac involvement in renal cell carcinoma is an uncommon but clinically important manifestation of metastatic disease. Presentations are variable and can easily be mistaken for common cardiopulmonary disorders, necessitating a high degree of clinical suspicion in patients with known malignancy. Accurate diagnosis depends on multimodal imaging, particularly cardiac MRI, which provides the most detailed assessment of myocardial involvement.

Management requires a tailored approach that accounts for disease extent, symptom burden, and the risks associated with surgery or systemic therapy. Although systemic treatments have improved outcomes for metastatic renal cancer, the prognosis for patients with cardiac metastases remains poor. Current understanding is based largely on isolated case descriptions, underscoring the need for collaborative studies and standardized reporting to better define risk factors, refine diagnostic pathways, and guide clinical decision-making in this rare scenario.
